# Adapting the eHealth Literacy Scale for Carers of People With Chronic Diseases (eHeals-Carer) in a Sample of Greek and Cypriot Carers of People With Dementia: Reliability and Validation Study

**DOI:** 10.2196/12504

**Published:** 2019-11-28

**Authors:** Areti Efthymiou, Nicos Middleton, Andreas Charalambous, Evridiki Papastavrou

**Affiliations:** 1 Department of Nursing Faculty of Health Sciences Cyprus University of Technology Limassol Cyprus; 2 Department of Nursing Faculty of Health Sciences University of Turku Turku Finland

**Keywords:** eHealth, literacy, scales, carers, technology, chronic disease

## Abstract

**Background:**

As the population ages, many more people will be in need of long-term care. According to a recent report by Alzheimer's Disease International and the Karolinska Institute, 84% of people with dementia are cared for at home and 16% in nursing homes. Several Web-based interventions have been developed to assist the work of carers at home. Measuring the levels of electronic health (eHealth) literacy is of top priority to facilitate inclusion of this population and develop training programs to enhance eHealth literacy skills.

**Objective:**

This study aimed to adapt the eHealth Literacy Scale (eHeals) for carers of people with dementia, who speak Greek as their native language and live in Greece and Cyprus, and to test the reliability and validity of the scale for carers.

**Methods:**

The content validity of the eHealth Literacy Scale for Carers of People With Chronic Diseases (eHeals-Carer) was assessed with an expert panel (N=10). A descriptive study with face-to-face interviews among 101 primary carers of people with dementia was conducted. In addition to the eHeals-Carer to assess their perceived eHealth literacy, participants responded to a brief questionnaire regarding characteristics of internet use and provided sociodemographic data. The internal consistency of the tool and the construct validity via an exploratory factor analysis (EFA) were explored.

**Results:**

The Mean Item-Level Content Validity Index (CVI) and Scale-Level CVI Average was 0.93. The participants were mostly women (75.2%, 76/101), aged less than 60 years (67.3%, 68/101) with secondary education. The internal consistency was estimated at a Cronbach alpha of .83. Two factors were extracted from the EFA: information seeking questions 1 to 5 (factor 1) and evaluation questions 6 to 8 (factor 2).

**Conclusions:**

eHeals-Carer is the first perceived eHealth literacy tool adapted for carers of people with dementia. The use of Web-based services available for carers could help them and improve the health care system in the long term. In Greece and Cyprus, there is a lack of services, and improving the digital skills of carers could provide them with the means to support themselves at home and improve care provision.

**International Registered Report Identifier (IRRID):**

RR2-10.2196/resprot.8080

## Introduction

### Background

As the population ages, old age diseases are on the rise, that is, many more people will be in need of long-term care in the years to come. In many countries, family and friends usually undertake the role of the carer filling the gap from the lack of organized health and social services, a phenomenon that is more common in Mediterranean and Eastern European regions [1].

According to a recent report by Alzheimer's Disease International and the Karolinska Institute, 84% of people with dementia are cared for at home and 16% in nursing homes [2]. Most carers of people with chronic diseases are aged older than 55 years, and women provide 71% of the annual informal care hours [3,4]. The global number of informal care hours is estimated to be around 6 hours per day or, on an annual basis, 82 billion hours of care. Carers experience stress, making them more vulnerable to infections and memory disorders, and they report a higher use of antidepressants and have high mortality rates [5-7]. The care of people with dementia can be rather demanding, as most patients may develop behavioral disorders in the course of the disease [8]. Carers search for information of the disease prognosis and treatment, services, and support as a way to manage the negative aspects of caregiving and use their social network, friends, families, health providers, and media (newspapers, television, and internet) to do so [9,10].

### Carers’ Pattern of Use of Web-Based Interventions and the Role of Electronic Health Literacy

Several Web-based interventions have been developed to assist the work of carers at home. They are easy to use and provide quick access to disease-specific information, as in the case of health care websites, psychoeducational platforms, applications, and telehealth and telemonitoring devices [11-13]. In most cases, these services have been provided only during the period of the research intervention, and no further information is provided on their use by carers [14]. According to Chiu and Eysenbach [15], a pattern of use of Web-based interventions made by carers is influenced by several factors such as accessibility, perceived effort, carers’ needs (personal skills, social support, carers’ beliefs, and years of care), and the style of use. In a modern framework developed to explain factors influencing the design of new technologies based on electronic health (eHealth) literacy level of the users, there is a discussion based on the individual characteristics (being a patient or a carer), the task dimension, and the experience using the technology [16]. Skills in searching, finding, appraising, and applying health information online have also been defined by Norman and Skinner [17], discussing eHealth literacy, which includes the following 6 literacies: traditional, information, media, health, scientific, and computer literacy. The latter 3 (ie, health, scientific, and computer literacy) are categorized according to the authors as context specific. This model has been modified and extended by other researchers [18-20], and a recent definition of eHealth literacy is provided by Bautista [21] and Paige et al [22]. eHealth literacy is redefined and “...involves the interplay of individual and social factors in the use of digital technologies to search, acquire, comprehend, appraise, communicate and apply health information in all contexts of healthcare with the goal of maintaining or improving the quality of life throughout the lifespan.” Taking the above into consideration, the individual characteristic, being a carer or a patient, may influence the person’s perceived eHealth literacy level. Low health literacy among carers of adults is associated with poorer health provision, care recipient health outcomes, and increased burden [23].

### Adapting the eHealth Literacy Scale for Carers of People With Chronic Diseases

There is a lack of published data on eHealth literacy level among carers of people with dementia and adapted or newly developed tools for this purpose.

Norman and Skinner [24] developed the eHealth Literacy Scale (eHeals) to measure the perceived skills that influence the eHealth literacy and consists of 8 items. It was originally tested among 664 adolescents, aged 13 to 21 years, in Canada and showed good metric properties. The scale is easy to administer. The items are short and incorporate a combination of the literacies presented in the Lily model, take no more than 10 min, and assess the way a person searches, assesses, and applies health information online. Even if there is a discussion concerning the lack of Web 2.0 questions [25], at present, it has been translated and used in many different languages and population groups. In the past 5 years, research studies seem to focus on the dimensionality and construct validity of the scale (eg, the number of factors the tool taps on) as well as other related variables such as internet access and use, computer skills, and determinants of eHealth literacy such as age, monthly income, health status, education, and chronic diseases [26-32].

The need for the eHeals to be adapted for the carers population as the eHealth Literacy Scale for carer of chronic diseases (eHeals-Carer), is associated with their caring needs. They usually search information for another person instead of for themselves and their personal health issues, and they are more receptive to technologies that assist them in their caregiving [33,34]. Adapting eHeals items to fit carers’ online style of use would facilitate their understanding of the topic and make the questions more comprehensible for their specific needs. This also facilitates their inclusion in the new technological era, as new online tailored services are increasingly provided to carers.

### Electronic Health Literacy Among Carers and Available Research in Greece and Cyprus

At the moment, we may only find information on the style of health-related internet use and possible predictors of this type of use made by carers [35,36].

In Greece, recently, a study identified older age and lower education among the main predictors of lower functional eHealth literacy in a Greek-speaking population [32]. We know that in Greece and Cyprus, the main reason for internet nonuse among older adults is the lack of skills [37,38]. In Greeks and Cypriots, among people aged 65 to 74 years, there is a decrease in internet use from 17.6% in 2012 to 11.1% in 2014 and from 12.7% in 2012 to 6.4% in 2014 for the age group of 75 to 99 years. On the basis of data from the *Internet in Cyprus* report, only 9.6% of the Greek Cypriots search the internet for health information on a weekly basis, and 43% of the sample has never searched the internet for health topics [38].

### Objectives

The aim of the study was 2-fold: (1) to identify available validated eHeals as part of a scoping review and (2) to evaluate the validity and reliability of the proposed eHeals for carers among a sample of Greek-speaking carers of people with dementia in Greece and Cyprus.

## Methods

### Literature Review on Available eHealth Literacy Scale Validations

As part of the validation process, we have searched following the methodology of a scoping review as described in the studies by Arksey and O’Malley and Peters et al [39,40] for relevant validations of eHeals to identify all possible alternatives regarding the different languages, population, statistics, and ratings and any available carers adapted version.

The main research questions of the review are as follows: (1) What type of statistical analysis is used to extract factors for eHeals? (2) How the Web 2.0 problem is handled in existing validations of eHeals? (3) Is there any difference in rating the scale? and (4) Is any eHeals validation for carers available?

We searched for all validations of eHeals in relevant databases (PubMed, CINAHL, MEDLINE, PsycINFO, and Scopus) and gray literature (eScholarship) until December 2018. Keywords used were eHeals and eHealth Literacy Scale.

The studies assessed are based on the following inclusion criteria: (1) the study should be related to the topic of eHealth literacy; (2) the study should be related to the scale reliability and validation; and (3) the study should be published in English

We did not include studies that used eHeals as a measure of eHealth literacy, but no information on validation was provided. The flowchart and related table of results are included in this paper as Multimedia Appendices 1 and 2.

### Validation Process of eHeals Carers in Greece and Cyprus

Following the literature review, we designed the validation and adaptation of the eHeals among Greek and Cypriot carers of people with dementia. Permission to use and adaptation of the scale were obtained by the authors [24]. The study followed the validation process as described by the World Health Organization following a double forward and backward translation strategy [41].

As part of the first step, we proceeded with the double forward and backward translation between the original English and Greek. Initially, 2 independent translators, both native speakers of Greek and fluent in English translated the scale into Greek. After comparing and merging the 2 translations into a single Greek translation by consensus, 2 independent back translations into English were derived by an additional set of 2 bilingual translators, 1 care professional and 1 researcher (ie, nurse trainer). In case of disagreement, we employed consensus meeting among the research team members based on expert opinion and existing literature.

In the second step, face validity by the research team followed. During this phase, researchers assessed the available Greek translation of eHeals and if the translated items corresponded to the English version of eHeals. The research team selected the final version in the Greek language and adapted it accordingly by adding a reference to the caregiving concept in every item of the scale. All items were modified accordingly to refer to the health and caregiving issues of a friend/relative, as, for example, in item 1: “I know what health resources are available” adapted to item 1: “I know what resources/information are available on the Internet concerning the health and caregiving issues of my friend/relative.” The caregiving issues on the scale are explained as the practical, financial, legal issues and information about the disease and available services. In the case of items 2, 3, and 4, we also added short clarification to facilitate understanding. Modifications of the scale are available in Table 1.

The content validity of the adapted items in the Greek language was assessed by a panel of experts in the field of eHealth and dementia or older people. Following this process, the questionnaire was piloted in 25 carers. Finally, the internal consistency of the final version of the Greek-adapted scale was tested among a sample of primary carers, and construct validity was followed with exploratory factor analysis (EFA).

**Table 1:** eHeals-Carer (eHealth Literacy Scale for Carers of People With Chronic Diseases) items: item difficulty, corrected item-total correlation, and factor loading.

Questions per factor		Mean (SD)	Median	Corrected item-total correlation	Factor loadings
**Factor 1**					
	Item 1: “I know what resources/information are available on the Internet concerning the health and caregiving issues of my friend/relative (practical, financial, legal issues, information about the disease and available services).”	3.51 (0.93)	4	0.48	0.485
	Item 2: “I know where to find helpful information on the Internet concerning the health and caregiving of my friend/relative (e.g. which websites I will search).”	3.35 (1.06)	4	0.59	0.540
	Item 3: “I know how to find helpful information on the Internet concerning the health and caregiving of my friend/relative (e.g concerning the process: google search).”	4.08 (0.82)	4	0.55	0.735
	Item 4: “I know how to use the Internet to answer my questions about the health and caregiving of my friend/relative (e.g how to ask in order to receive a proper reply to my question).”	3.83 (1)	4	0.53	0.656
	Item 5: “I know how to use the information about the health and caregiving of my friend/relative I find on the Internet to help me (practical, financial, legal issues, information about the disease and available services).”	3.75 (0.85)	4	0.55	0.500
	Total	18.49 (19)	19	—^a^	—
**Factor 2**					
	Item 6: “I have the skills I need to evaluate the resources/information I find on the Internet concerning the health and caregiving of my friend/relative.”	3.70 (1.05)	4	0.59	0.756
	Item 7: “I can tell high quality resources/information from low quality resources/information on the Internet concerning the health and caregiving of my friend/relative.”	3.75 (1)	4	0.59	0.731
	Item 8: “I feel confident in using information from the Internet to make decisions concerning the health and caregiving of my friend/relative.”	3.30 (1.08)	3	0.57	0.595
	Total	10.77 (2.62)	11	—	—
Total scores from both factors		29.27 (5.30)	29	—	—

^a^Not applicable.

### Recruitment

#### Recruitment Panel of Experts for the Content Validity Index

To proceed with the content validity index, we invited 10 experts to reply to the content validity of the questionnaire. The experts were invited because of their work on eHealth and/or dementia domain. Of 10 experts, 8 were health professionals: 3 health care professionals, nurses, and psychologists working in the field of technology (robotics and digital literacy of older people), 1 member of the Greek team of the European Health Literacy Survey, and 4 health care professionals working in dementia care. The remaining 2 were information technology experts working in the field of eHealth.

#### Recruitment of Primary Carers

The data collection of primary carers was made in the framework of the research protocol for “the Association of Health Literacy and Electronic Heath Literacy with Self-Efficacy, Coping and Caregiving Perceptions Among Carers of People with Dementia: Research Protocol for a Descriptive Correlational Study” [42].

The final sample of the protocol was estimated with 95% power and a type 1 error of 5% to 168 primary carers. All questionnaires were pilot tested in 25 primary carers of people with dementia [43].

The validation of eHeals adapted for carers proceeded with a convenience sample of 101 carers from Greece and Cyprus, based on the subject-to-item ratio 10:1 [43-45]. Participation in the study was voluntary, and the recruitment of the sample lasted for 1 year. Eligibility criteria were broad and included being a carer of a person with dementia, speaking Greek, and being aged older than 18 years. Researchers approached carers at Dementia Day Care Centers in Athens, Greece, and Limassol, Cyprus, or during training courses and public awareness campaign events directed to carers of people with dementia. In the case of Dementia Centers, the scientific supervisors assisted the researcher to arrange the appointment at the time of the day that carers were available. In the case of public events, the researcher distributed leaflets, and carers expressed their interest in participating. The researcher arranged a face-to-face survey appointment to administer the questionnaire.

### Measures

The measures were as follows:

- Content Validity Index [46]: all expert panel participants received the questionnaire adapted for carers in the Greek language and assessed item phrasing, simplicity by commenting on every item and relevance on a 4-point scale: not relative, somehow relative, quite relative, and relative.
- Carers replied to the Greek version of eHeals-Carer, which includes 8 items, each with a 5-point response scale from 1 (strongly disagree) to 5 (strongly agree). As shown in Table 1, all 8 items were adapted accordingly to specifically refer to the caregiving role.
- Carers also provided the following basic sociodemographic information: gender, age, education level (based on the international standard classification of education), employment status, carers’ relationship, living status, and being supported by a secondary carer or not), and replied to a series of questions with regard to internet use, either personal or dementia-specific online use. As part of the sociodemographic information, we have used a visual analog scale for measuring the socioeconomic position, Ladder questionnaire [47,48]. The participants were asked to assess where they stood on a ladder in comparison with other people in Greece or Cyprus, given that in the bottom of the scale are the people with the worst profession or unemployment, least money, and lowest education.

### Data Analysis

In content validity, we reported the following 3 indexes: (1) Mean Item-Level Content Validity Index (Mean I-CVI), measuring the proportion of relative and very relative responses of the items; (2) Scale-Level Content Validity Index Average (S-CVI/Ave), measuring the average score of the responses of quite relevant and very relevant of every expert; and (3) the Scale Content Validity Index Universal Agreement (S-CVI/UA), measuring all items that all raters assessed as quite or highly relative. As scale CVI, we usually consider the S-CVI/Ave because the S-CVI/UA decreases as the number of raters increases [46].

The internal consistency of the scale was assessed with a Cronbach alpha, and the dimensionality of the scale was explored with EFA. This was the first time that the scale was validated in Greek among carers, and dimensions were not hypothesized before the validation. Confirmatory factor analysis (CFA) will be calculated with the total sample of the study protocol based on the EFA findings.

### Ethical Consideration

The Cyprus National Ethical Committee (EEBK ΕΠ 2016.01.151) and the Cyprus Commissioner for Personal Data Protection (3.28.460) approved the study. As the study was conducted in 2 countries, the study protocol also received approval by the Scientific Committee of Alzheimer Athens Association (March 17, 2017).

## Results

### Results of Literature Review on Available eHealth Literacy Scale Validations

According to the first step of the validation process, we conducted a review to identify all possible eHeals validations to decide on the methodology and avoid any replication of existing measures for this specific population.

The scale has been validated and adapted in many different languages and population groups, using either convenient sample recruitment strategies or randomized recruitment techniques (as random telephone dialing). In the last 3 years, the validation studies of the specific tool were increased, showing a tendency toward eHealth literacy research. Only in 1 study from Slovenia did we find the validation of an extended version of 20 items (6 factors) including the Web 2.0 parameter as discussed earlier by Norman [49,50]. In 21 cases, the authors preferred a combination of the original scale adding questions to assess health-related internet use and internet use in general [17,25,27-29,31,51-64]. The reliability in the majority of the studies was quite high, that is, over 0.80. The lowest reliability was presented in a student sample in Bangladesh and in the 6 dimensions of the Slovenian version [50,61]. In 6 of 26 studies, the sample recruitment focused on older adults [25,52,59,65-67].

A series of studies have identified or confirmed the unidimensionality of the eHeals [25,30,31,57,68-70]. However, the latest studies seem to propose either a 2-factor model or a 3-factor model [27-29,52,54,59,62,67]. The study by Soellner et al [64] was one of the first to propose a 2-factor model with an information seeking (questions 1-5 and 8) and an information appraisal (questions 6 and 7) component. This model was later confirmed by Diviani et al [28]. Subsequent studies also supported a 2-factor model, yet with a different set of questions, for example, the first 4 questions tapping on factor 1 and the last 4 questions on factor 2 [27,29]. With regard to the 3-factor model, the most commonly accepted dimensions are as follows: awareness (questions 1 and 2), skills (questions 3-5), and evaluation (questions 6-8). Paige et al [63] proposed a 3-factor model with a different categorization, which, instead of skills and evaluation, includes information seeking (questions 3 and 4) and information engagement (questions 5-8).

In almost all cases, the scoring system distinguished between high and low scores without providing information for a medium level. In 12 papers, the level was calculated by summarizing all items, and in 4 validation studies, the level was calculated by summing up all items and dividing the score with the number of the scale or of the factor. The highest score of eHeals among the studies included in this review is presented in the study by Chung and Nahm [65] for a sample of 886 adults, with a mean age of 62 years and eHeals literacy mean score of 30.94 (SD 6).

In 5 studies, the researchers used a principal component analysis (PCA), in 11 cases EFA, in 8 studies CFA, and in 3 studies either PCA or EFA and then CFA (Multimedia Appendix 2). In 4 studies, they followed item response theory and Rasch modeling.

This review provided the basis for our validation study. On the basis of the above results, the discussion for the use of classical test theory and item response theory in behavioral and social science [71], and the aim of our study (to adapt an already developed short scale), we decided to follow the classical test theory validation and the use of EFA. As there were many available validations providing different dimensions, we decided to explore the dimensions in this target group and confirm these factors in a larger study sample of carers.

Our decision to adapt for a specific population was in accordance with the measurement modifications for diverse populations [72]. The reasons for modifying this scale were as follows: (1) carers were a different population from the one that participated in the development of the original scale; (2) the scale lacks the caregiving concept that carers would be related to; and (3) if the eHeals was used as it is, there might be a misinterpretation of the items through the caregiving filter. To proceed with the adaptation of the eHeals, we followed an extensive literature review on the eHealth literacy research among carers and older people. Carers’ research on eHealth literacy was limited, but we encountered valuable information on the internet use among carers of frail older people and people with dementia. On the basis of this research, we were able to understand how carers may use the internet in relation to caregiving. They mostly searched for disease-specific information, services for the patients, practical issues, and legal and financial issues and to communicate through emails and chat sites [73-75]. In this regard, we decided to proceed with the context-specific modifications of the eHeals as has been discussed in the following subsections.

### Content Validity of eHealth Literacy Scale Carers in Greek

Mean I-CVI and S-CVI/Ave was 0.93 in both cases. S-CVI/UA was 0.60.

Experts made no further comment on the phrasing of the scale, apart from 3 comments on 3 different items (items 1, 2, and 9), that did not change the final meaning of these items.

### Demographic Information of Primary Carers

As part of the reliability and construct validity, our sample comprised primary carers, mostly women (75.2%, 76/101), caring for their parents (61.3%, 62/101) living in the same household (61.3%, 62/101), aged younger than 60 years (67.3%, 68/101), having completed 12 years of education or more (92.0%, 93/101), mostly unemployed or pensioners (62.3%, 63/101), and receiving assistance from a secondary carer (78.2%, 79/101). Detailed demographics are presented in Table 2. Socioeconomic position was assessed with the use of the ladder figure questionnaire with 10 steps, providing a mean score of 5.8.

**Table 2:** Demographic information of the carers sample (N=101).

Characteristics		Value, n (%)
**Gender**		
	Women	76 (75)
	Men	25 (25)
**Age (years)**		
	<59	68 (67)
	60-79	33 (33)
	>80	0 (0)
**Education**		
	No primary education (ISCED^a^, level 0)	0 (0)
	Primary education (ISCED, level 1)	8 (8)
	Secondary education (ISCED, levels 2-4)	54 (53)
	Tertiary education (ISCED; levels 5.1, 5.2, and 6)	39 (39)
**Employment status**		
	Employed	38 (38)
	Unemployed (including pensioners)	63 (62)
**Carers’ relationship**		
	Caring for parent	62 (61)
	Caring for spouse	28 (28)
	Caring for other (relative/friend/neighbor)	11 (11)
**Secondary carer support**		
	Yes	79 (78)
	No	22 (22)
**Living status**		
	Together with person with dementia	62 (61)
	Living in other’s house	39 (39)
**Most frequent internet use for carers**		
	Search of information	40 (43)
	Reading news	15 (16)
	Entertainment (movies and music)	12 (13)
	Social networks	8 (9)
	Emails	9 (10)
	Professional reasons	8 (9)

^a^ISCED: International Standard Classification of Education.

### Internet Use Characteristics

Of 101 participants, 92 used the internet with the more frequent reason of private internet use: *searching for information on different topics*. Of all participants, 97.0% (98/101) visited websites; 76.2% (77/101) used social networks, such as Facebook, Twitter, and LinkedIn; 81.1% (82/101) used email to communicate; 83.1% (84/101) used interactive services (eg, Viber, Skype, forums, and chatrooms); and only 42.5% (43/101) accessed electronic learning (eLearning) courses.

In the questions regarding online search of dementia-specific information such as disease information, practical issues, legal information, and available services, almost all participants 90.0% (91/101) stated that they had accessed online dementia resources and mostly websites. Almost half of the participants (40.5%, 41/101) had used social networks, and 42% (42/101) had used email to communicate and searched for information with other carers, family, and health professionals. The use of interactive services and eLearning courses were the least preferred resources to communicate and receive information or training with 32.6% (33/101) and 12% (11.8/101) users equivocally.

Among all participants, 51.4% (52/101) used a mobile phone to access information for dementia care or to communicate with other carers or health care professionals. Adding to the above result, of 52 participants who have used the internet on their mobile phone, 86% (45/52) have accessed websites, 54% (28/52) accessed social networks, 39% (20/52) used emails, 42% (22/52) used other interactive services, and 5% (3/52) used eLearning services through their mobile phone.

**Reliability**

Internal consistency of the scale was measured with Cronbach alpha of .83. All items appeared important with item-total correlations ranging between .48 and .59. In all cases, the Cronbach alpha was lower if any of the items was removed.

**Figure 1:**
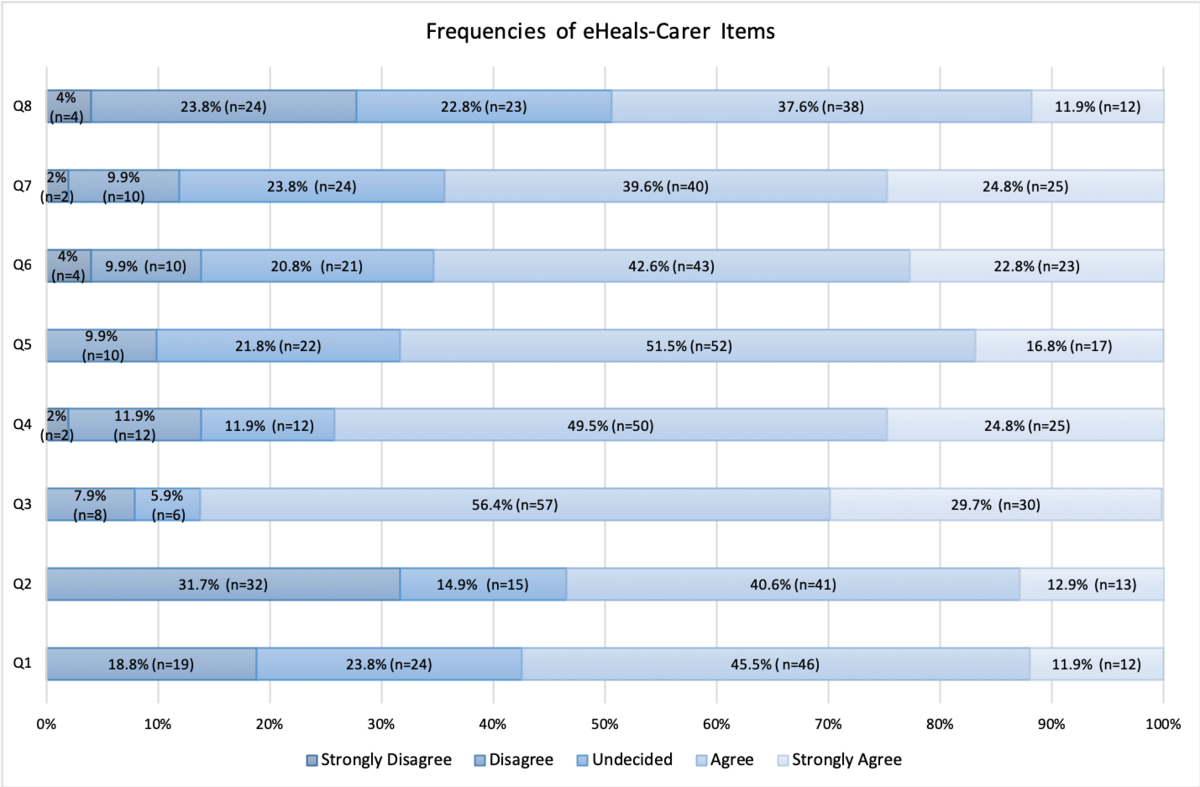
Frequencies of responses of eHeals-Carer (eHealth Literacy Scale for Carers of People With Chronic Diseases) items.

The items with the highest frequency of replies of agreement (agree and strongly agree) were item 3 “I know how to find helpful information on the Internet concerning health and caregiving of my friend/relative (e.g. concerning the process: google search),” item 4 “I know how to use the Internet to answer my questions about the health and caregiving of my friend/relative (e.g. how to ask in order to receive a proper reply to my question),” and item 5 “I know how to use the information about the health and caregiving of my friend/relative I find on the Internet to help me (practical, financial, legal issues, information about the disease and available services).” Item 8 “I feel confident about using information from the Internet to make decisions concerning the health and caregiving of my friend/relative” had the lowest scores of agreement (Figure 1). This was also confirmed by mean scores of every item of the scale as presented in Table 1. The total mean score of the scale eHeals-Carer was 29.27 (SD 5.30).

### Construct Validity

The dimensionality of the scale was explored in EFA, principal axis factoring with Varimax rotation. Kaiser-Meyer-Olkin measure sampling adequacy was 0.80, and the Bartlett test of sphericity was statistically significant (χ^2^_28_=261.5 *P*<.001). Overall, 2 factors with eigenvalue greater than 1 were extracted, with the first factor explaining 24% of the variance and the second factor 23% (rotation sums of square loadings). After Varimax rotation, a clear structure was revealed with no cross-loadings. Items 1 to 5 loaded on the first factor and seem to tap on the *information seeking* aspect of eHealth literacy. Items 6 to 8 loaded on the second factor and tapped on the *evaluation* aspect of eHealth literacy. Reliability analysis for factor 1 provided a Cronbach alpha of .77 (mean 18.48 [SD 3], median 19), and for factor 2, a Cronbach alpha of .78 (mean 10.77 [SD 2.62], median 11).

## Discussion

### Principal Findings

We searched the literature to identify all possible validations of the eHeals and to check if there was any adapted version for this population. We adapted and validated the scale for carers, resulting in a scale with high Mean I-CVI (0.93) and high reliability (0.83). The data analysis supported 2 factors: information seeking and evaluation. The first factor includes the 5 items of eHeals 1 to 5, and the second factor includes 3 items 6 to 8. In the literature, we identify different categories derived from the analysis of eHeals including awareness (1 and 2), skills (3-5), information seeking (1-5 and 8 or 3-4), information appraisal (6 and 7), information engagement (5-8), and evaluation (6-8). We have also identified 2 factors related to seeking and appraisal skills as in the case of Soellner et al [54], but with a different combination of the eHeals items for the 2 dimensions. This difference, from other researchers, might derive from the cultural adaptation of the tool. In item 5 “I know how to use the information about the health and caregiving of my friend/relative I find on the Internet to help me (practical, financial, legal issues, information about the disease and available services)” was perceived as a competence/skill item on how to do rather than as an item for evaluating the information.

In eHeals, as initially developed by Norman and Skinner, more than 1 literacy is included per item of eHeals [17,53]. For example, traditional, information, computer, and health literacy are included in all items of the scale. Media and scientific literacy can be identified in the evaluation subscale [53]. We adapted the short-scale 8-item eHeals for carers to investigate carers’ eHealth literacy levels. In this adaptation, we consider the different needs of carers regarding health and eHealth literacy skills. According to a recent scoping review, carers’ levels of health literacy are considered adequate, even if they largely depend on the scale used [23]. Carers are the people who manage the communication with the health care providers and the care recipient, manage support services for the dependent person, and make health-related decisions. We also know from previous studies that carers’ health literacy levels and eHealth literacy skills may vary according to the person’s characteristics: being a carer or not, as this has been identified for the health-related internet use in this population [36]. Carers report higher levels of health literacy in comparison with the care recipients [23]. They usually search for health-related information for the cared-for person and use the internet to find information about the disease prognosis and treatment, legal and financial issues, practical issues, and communication [34,36,73]. Online information and services are important for the health self- management [9]. This is also confirmed in a study by Anderson et al and the analysis of 2345 carers’ posts in 9 websites. Researchers have categorized posts in 4 topics: social support—communication and inclusion, search of information, sharing of memories with the person with dementia, and sharing information with other carers [76].

In Greece and Cyprus, carers are the core element in the care provision of people with dementia, covering the lack of tailored services by the National Health System [77,78]. The development of eHealth tools has been promising in this area, assisting carers in everyday tasks, but still much needs to be done to increase the use of these tools by carers. As a first step, we need to investigate the eHealth literacy levels of carers by using a short, easy-to-comprehend tool. In this study, we adapted the eHeals questionnaire to mirror the carers’ role as an effort to provide this adapted tool to carers in Greece and Cyprus. In Greece, Xesfingi and Vozikis [32] assessed the eHealth literacy level in a sample of 1064 citizens, ages ranging from 15 years to older than 80 years, with older people and the less educated to be less eHealth literate. In Cyprus, there is no available literature measuring eHealth literacy levels among older people or carers.

We consider that this scale assists in the assessment of eHealth literacy level of carers in 2 ways. Firstly, in practice, as the health care system, not-for-profit organizations, and academic institutions could develop tailored programs for the online needs of the carers. In this way, carers may improve the way they access and evaluate dementia-specific information or information regarding their health. Secondly, in research, as we provide a validated tool for use in future studies investigating the determinants of eHealth literacy, its association with the burden and other aspects of the caregiving role, as well as a process outcome measure in intervention studies targeting eHealth literacy. In this way, eHealth inequalities may be decreased, as carers improve the management of the disease and their burden because of a better use of the available Web-based services.

Finally, through the validation process in this diverse population, we identified culturally specific issues related to the understanding of the items of the first-dimension *seeking information*, and we consider important in future research on the development and validation of eHealth literacy tools that researchers include short exemplars to facilitate understanding of the *how to* items when related with internet users’ skills.

### Limitations

Carers of people with dementia in this study are considered a convenient sample. Participation rate did not exceed 31% as revealed in the piloting phase of the study protocol. Carers in Greece and Cyprus were not easy to identify if they had not attended a dementia center. As a consequence, the final sample included in this validation was small. The study should be repeated in a larger sample, among carers of patients of other chronic diseases and could be used for cross-country comparisons between Greek and Greek-Cypriot carers.

Even if the eHeals has been adapted for carers, no item about Web 2.0 has been added in the 8-item scale. We only added it in the supplementary section of the internet use characteristics [49]. Carers use the internet to interact with health care professionals and other carers [79-81]. This type of internet use (interaction with social networks: forums and chatrooms) is not depicted in this scale, making this adapted version limited but convenient for use in large study protocols when there is a need of a short tool with high reliability and validity for measuring eHealth literacy among carers.

### Conclusions

The validation of eHeals-Carer provides the first questionnaire measuring perceived eHealth literacy skills adapted to carers. At the moment, there is no other scale measuring eHealth literacy levels for carers available. The development of new tools on eHealth literacy measuring functional aspects adapted to specific needs seems to be the next step in this research area. Carers of people with dementia, in the majority, are people aged older than 50 years, children, or spouses, with low use of care-specific Web-based services. The use of the online services available for carers could facilitate the carers and the long-term health care system. In Greece and Cyprus, there is a lack of services for carers, and by improving their digital skills, we could provide them with the means to support themselves and improve the care they provide. With the increased offer of Web-based services tailored for carers, the improvement of their digital skills will become more demanding in the years to come. Furthermore, public and private services in Greece and Cyprus are updating their service systems to be following technological progress. In this era, carers can be included if we provide them with adequate and appropriate eHealth literacy training programs.

## Appendix

Multimedia Appendix 1 Scoping review flowchart.

Multimedia Appendix 2 Scoping review validation results of eHeals (Electronic Health Literacy Scale).

## Abbreviations

CFA: confirmatory factor analysis
CVI: content validity index
EFA: exploratory factor analysis
eHealth: electronic health
eHeals: eHealth Literacy Scale
eHeals-Carer: eHealth Literacy Scale for Carers of People With Chronic Diseases
I-CVI: Item-Level Content Validity Index
PCA: principal component analysis
S-CVI/Ave: Scale-Level Content Validity Index/Average
S-CVI/UA: Scale-Level Content Validity Index/Universal Agreement
