# Supplementary figures and images for: Adapting the eHealth Literacy Scale for Carers of People With Chronic Diseases (eHeals-Carer) in a Sample of Greek and Cypriot Carers of People With Dementia: Reliability and Validation Study
Source: J Med Internet Res. 2019 Nov 28;21(11):e12504. doi: 10.2196/12504 (PMC6908974; doi:10.2196/12504)

Multimedia Appendix  
Scoping Review Flow Chart

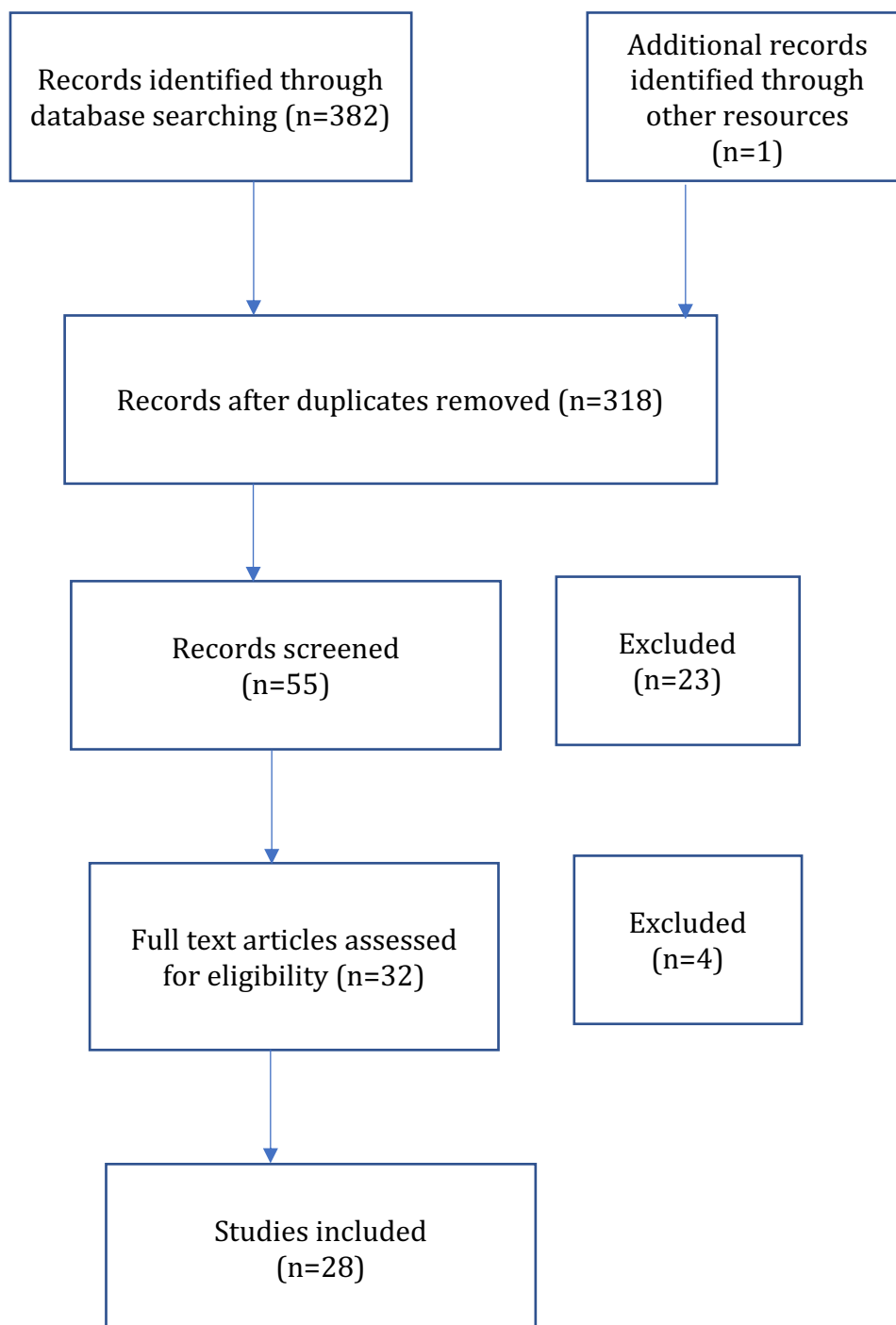

Supplement: Multimedia Appendix 1 [file jmir_v21i11e12504_app1.pdf]
