# Supplementary material for: Adapting the eHealth Literacy Scale for Carers of People With Chronic Diseases (eHeals-Carer) in a Sample of Greek and Cypriot Carers of People With Dementia: Reliability and Validation Study
Source: J Med Internet Res. 2019 Nov 28;21(11):e12504. doi: 10.2196/12504 (PMC6908974; doi:10.2196/12504)
Supplement: Multimedia Appendix 2 [file jmir_v21i11e12504_app2.pdf]

Multimedia Appendix "Scoping review results of eHeals"

	Authors	number of items	aim	language	reliability	dimensions	sample	recruitment process	scoring
1	Norman & Skinner, 2006a [24]	8 items (+ internet use questions), self-administered	to assess eHealth literacy in wide population	Engllish	Cronbach alpha =.88,intra-class correlation in test retest reliability = .49 (modest stability over time)	1 factor (PCA)	664 adolescents (age 13-21)	single session randomised intervention trial	5-point Likert scale, perceived skills (high - low reference not specific categories)
2	Van Der Vaart et al, 2011 [25]	8 items (+ in study 1, general and health related internet use and in study 2: general internet use and performance test), administration way not defined	reliability and construct validity of a Dutch version	Dutch	Study 1: Cronbach alpha=.93 Study 2:Cronbach alpha= .92	Study 1 &2: 1 factor (PCA)	Study 1: 227 people with rheumatic diseases, mean age 52, Study 2: 88 people, general population, mean age 43	Study 1: randomised sample by patient database Study 2: random recruitment by random dialing numbers	5-point Likert, scale, Study 1: mean sum score: 28.2 , SD=5.9 tudy 2: mean sum: 27.6, SD=5.9
3	Koo et al (2012) [30]	8 items, self-administered	validation of eHeals to Chinese school children	Chinese	Cronbach alpha=.92, assessment of concurrent validity	1 factor (PCA)	219 six graders School children	single group cross sectional study, part of a larger study	5-point Likert scale , total score: 8 to 40 , mean score 28.4, SD=7.6)
4	Mitsutake et al, 2011; Original Paper in Japanese [70]	8 items, self-administered	N/A	Japanese	Cronbach alpha=.93	1 factor (CFA)	3000 participants	randomly selected	5-point Likert scale, total score: 8 to 40,
5	Neter & Brainin, 2012 [31]	6 items (+ internet access, digital literacy, health information sources, content, search strategies, evaluation criteria, perceived outcomes of health information search, perceived health), telephone interview	Assess levels of eHealth literacy among Israeli random population	Israeli	Cronbach alpha=.86	1 factor (EFA/PCA)	4286 adults	Random digital-dial telephone survey	5-point Likert scale, mean 3.34 (SD .88), 2 groups (high level >3.4 and low level <3.4)
6	Soellner et al, 2014 [54]	8 items (+ subscales of the Health competences questionnaire, self-efficacy scale, internet use as information source and time spent online), self-administered, paper and pencil survey	Validation of eHeals in German among adolescents	German	Cronbach alpha for dimension information seeking: .88 and for information appraisal .83	2 dimensions: information seeking (1-5 & 8) and information appraisal (6 & 7) (CFA)	327 students with mean age 18.10	administered as part of a larger study, cross sectional paper-pencil survey, Grade 12 class sessions in 4 Gymnasia in Cologne	5-point Likert scale , Factor 1: 3.57 SD= .78, Factor 2: 3.70 SD= .96
7	Paramio Perez et al, 2015 in Spanish [69]	8 items scale (+ well-being scales), self-administered	validation in Spanish	Spanish	Cronbach alpha=.87	1 factor (EFA)	447 university students	Convenience sample	5-point Likert scale, total score: 8 to 40, high and low scores
8	Park & Lee, 2015 [55]	8 items (+internet use and 2 supplementary items), self-administered online	Assess levels of undergraduate nursing students in South Korea	Korean	Cronbach alpha= .86	n/A	176 nursing undergraduate student between age 20-30	Convenience sample	5-point Likert scale, total score: 8 to 40, mean 27.06 SD=4.2 (high over 27 and low below 27)
9	Saffarzadeh, Areo, 2015 [53]	8 items (+ health related internet use, internet use) self-administered paper and pencil	to explore health related internet use (use of high quality of websites and quality of the physician), the internal	English	Cronbach alpha= .94, Cronbach alpha of items 3-5= .93, Cronbach alpha of items 6,7)= .88, Cronbach alpha of (items 8,9,10)= .82	1 Factor (PCA) 2 & 3 factors (Varimax rotation factor analysis)	79 patients or their caregivers of Otoryngology head and Neck Surgery	Convenience sample	5-point Likert scale , 0-100 scale tranformation, mean 66.3 (SD= 20.5)
10	Chung & Nahm, 2015 [65]	8 items, self-administered	Validate eHeals for older adults	English	Cronbach alpha=.94	1 factor (EFA)	866 adults (mean age 62.8)	Original sample from Bone Power study. Online recruitement from Senior health and healthyVet	5-point Likert scale , total score: 8 to 40, mean 30,94 SD=6
11	Bazm et al, 2016 [56]	8 items (+ 10 questions to assess computer and internet skills), self-administered	Validation of the Iranian version of eHeals	Persian	Cronbach alpha=.88	1 factor (PCA)	525 youth people	Randomly selected	5-point Likert scale , total score: 8 to 40, mean score not available

12	Nguyen et al, 2016 [68]	8 items, self-administered	to investigate the eHeals properties with RASCH modelling	English	Study 1: Person reliability (equivalency of Cronbach alpha= .80. Study 2: Person reliability= .81	study 1 & 2: 1 factor (EFA) Rasch modeling (rating scale analysis)	Study 1: 164 undergraduate students (18-34- 83.6%: 20-21) Study 2. 366 individuals 59% aged 18-32 years	1. convenience sample 2. acquired by Amazon's Mechanical Turk (Mturk)	5-point Likert scale, total scroe: 8 to 40, no mean score available
13	Caro et al, 2016 , only abstract available [57]	8 items (+ 2 scales of self-esteem and life satisfaction), self-administered	validate in Italian	Italian	Cronbach alpha=.87, Test retest correlation=.78	1 factor (EFA)	650 university student (age 18-45)	N/A	5-point Likert scale, total scote: 8 to 40, high and low scores
14	Tubaishat & Habiballah, 2016 [58]	8 items (+ perceived level of internet skills, frequency of internet use, perceived usefulness of internet on health decisions, importance of health related internet access), self-administered	Assess the levels of ehealth literacy among nursing students in Jordan	Arabic	Cronbach alpha=.81	N/A	541 nursing students	Discriptive cross sectional sample, Convenient sample	5-point Likert scale , high and low scores, mean= 3.62 SD=.58
15	Paige et al, 2017 [67]	8 items scale, self-administered web based	Explore unidimensionality (1 factor) and reliability of the eHeals scale	English	Classical test: Cronbach alpha=.90, item response theory technique	3 factor model (CFA) , 70% explained by factor 1, 9% and 5% by 2 and 3 and Partial Credit Model (PCM)	811 participants of online survey with cardiovascular disease, arthritis, mental health disorder, chronic lung disease and cancer	web based survey	5-point Likert scale , total score: 8 to 40, high and low scores, (mean =30.34 SD=5.30)
16	Stellefson et al, 2017 [59]	8 items (+perceived health status and experience with social media platforms to access and share health information), self-administered	aiming to validate telephone version of eHeals for older adults, investigae 2 and 3 factor models	English	exploratory structural equation modeling, and IRT analysis	Exploratory structural equation modeling (PCM) : 3 factor model (2 of the 3 factors are correlated and provide evidence for unidimensional structure for older adults	283 older adults	telephone survey: random digital dialing as part of Florida Consumer Confidence index	5-point Likert scale, total score: 8 to 40, high and low scores, mean= 29.05 SD= 5.75
17	Diviani et al, 2017 [28]	8 items (+ general and health related internet use), self-administered web based	validate in Italian	Italian	Cronbach alpha= .89 (Classic theory and Rasch modeling)	2 factor solution (PCA & CFA)	296 Italian speaking region of Switzerland	2 surveys (summer 2013 and Spring 2015	5-point Likert scale , total score: 8 to 40, high and low scores, (mean =26.65 SD= 6.28), study 1: 27.21 SD= 6.083 and study 2: 26.27 SD= 6.388)
18	Sudbury-Riley et al, 2017 [52]	8 items (+information and resources to cover the web 2.0 with use of social media, self-administered online	measurement invariance: measuring the same traits in different groups and investigate a 3 factor model	English (UK, new Zealand, USA)	USA Cronbach alpha=.92, uk.93, new Zealand=.91	3 factor structure: awareness (1,2), skills (3, 4,5) and evaluation (6, 7, 8), CFA	996 baby boomers	random sample of 3 countries	5-point Likert scale, table with mean item scores
19	Dashti et al, 2017 [27]	8 items (+ websites for health related information, frequency of internet use), self-administered	Assess the level of ehealth literacy among Medical science students, validation of the tool	Persian	CVI (10 experts)Cronbach alpha = .89	2 categories: Q1,2,3,4 AND Q 5,6,7,8 (EFA)	192 Medical students	Convenient sample	5-point Likert scale , total score: 8 to 40, high and low scores, (mean =28.21 SD= 6.95)
20	Richtering et al, 2017 [60]	8 items (+ Health Literacy Questionnaire), self-administered online	Assess levels of eHealth literacy and health literacy among population with high cardiovascular risk	English (Australia)	Rasch Modeling, PSI (internal constancy: 0.90)	Unidimensionality is not well supported- probable 2 concepts	392 participant of CONNECT study , a randomized control trial	Randomized sample	5-point Likert scale , total score: 8 to 40, high and low scores, mean=27.1 SD=6.67
21	Islam et al, 2017 [61]	8 items (+ computer knowledge and internet use, use of Web 2.0 for health information and perceived health), self-administered	eHealth literacy levels in Southeast Asia	English (even If not clearly stated0	Cronbach alpha=.074	1 factor (EFA)	199 students in Bangladesh	Convenient Sample	5-point Likert scale , total score: 8 to 40, high and low scores, 5 point Likert scale (2 reverse questions)

22	Petric et al, 2017 [50]	20 items extended version (+ users' activities), online self administration	eHealth literacy level among online health communities and Development of extended eHeals	Slovenian	Validating information (Cronbach alpha=.75), understanding information (Cronbach alpha=.81), awareness of sources (Cronbach alpha=.80), perceived efficiency (Cronbach alpha=.75), recognizing quality (Cronbach alpha=.52) and being smart on the net (Cronbach alpha=.70)	6 dimensions (EFA and CFA)	644 users (mean age 40 years0	Random sample, MedOver Net users	5-point Likert scale (2 reverse questions), dimension 1: mean=3.80 SD= .61 dimension 2: mean=3.11 SD= .75, dimension 3: mean=3.98 SD= .67, dimension 4: mean=3.94 SD= .65, dimension5: mean=3.84 SD= .80, dimension 6: mean=3.74, SD=0.78)
23	Aponte & Nokes, 2017 [66]	10 items (+ focus groups), paper pencil administration	To assess the internet use and eHealth literacy of older Hispanic with Diabetes 2	Spanish (USA)	Cronbach alpha=. 98	1 factor (EFA)	20 Hispanic adults with Type 2 diabetes(mean age 74 years)	Senior Center in East Harlem, Convenience Sample, Mixed method design	5-point Likert scale, range 8 to 40, Mean score=22.35 (SD= 12.96)
24	Hyde et al, 2018 [62]	7 items scale (item 3 removed) (+ internet characteristics), elf-administered web based	verify 3 factor model structure	English	Factor 1: Composite reliability: .89, factor 2: . 92 and factor3 .89	3 factor structure: awareness (1,2), skills (3, 4,5) and evaluation (6, 7, 8) (CFA)	256 MRI and CT outpatients	convenient sample, MRI and CT outpatients	5-point Likert scale, no mean score available
25	Paige et al, 2018 [22]	8 items scale (+ health related internet use), web-based self-administered	invariance measurement, better model fit	English (US)	Factor1. Cronbach alpha =.84, Factor 2, Cronbach alpha=.88, Factor 3 Cronbach alpha=.84	1-4 factors structure (CFA). Better fit=3 factor model: information awareness (1, 2), information seeking (3,4), information engagement (5,6,7,8)	829 adults: millennials, generation X, baby boomers, silent generation	stratified by race (Caucasian, black/African)	5-point Likert scale , Factor 1(mean =7.48 SD= 1.71), Factor 2(mean 7.85 SD=1.51), Factor 3 (mean= 14.89 SD=2.88)
26	Gazibara et al, 2018 [29]	8 items scale (+ age of 1st internet use), self-administered	validate in Serbian and evaluate eHealth literacy	Serbian	Cronbach alpha=.85	1st factor: Q1,2,3,4 and 2nd factor: Q 5,6,7,8 EFA	702 students	4 high schools in Belgrade (randomly selected)	5-point Likert scale, total score: 8 to 40, high and low scores, mean 26 (20-30)
27	Chang & Schulz 2018 [51]	8 items scale (+ 10 item scale of media and computer literacy and 9 items on the use of information), web based self administered	development of eHe	Chinese	Cronbach alpha=. 95	1 factor (EFA and CFA)	352 interviews	Convenient sample of Public Hospital, Ethinic minority of Han, mandarin speakiing	5-point Likert scale, mean score is estimated per group (age, sex, education , resident, occupation and income)
28	Chung et al, 2018 [64]	8 items scale (+ internet use behaviour), elf-administered web based	validation in Korean	Korean	CVI, Cronbach alpha. Scale CVI .83, item CVI >.67	1 factor (EFA)	500 young adults from Korean internet survey panel	Korean internet survey panel	5-point Likert scale , total scote: 8 to 40, high and low scores, (mean 28.06 SD 4.81)

a. PCA= Principal Component Analysis, b. CFA= Confirmatory Factor Analysis, c. EFA= Exploratory Factor Analysis, d. Qn= Question, e. IRT= Item Response Theory, f. PCM=Partial Credit Model, g. UK=United Kingdom, h. USA=United States of Amerika, i. CVI= Content Validity Index

This is a Multimedia Appendix to a full manuscript published in the J Med Internet Res. For full copyright and citation information see http://dx.doi.org/10.2196/jmir.12504
